# Ovarian Epithelial Cancer Stem Cells

**DOI:** 10.1100/tsw.2011.112

**Published:** 2011-06-09

**Authors:** Irena Conic, Irena Dimov, Desanka Tasic-Dimov, Biljana Djordjevic, Vladisav Stefanovic

**Affiliations:** University School of Medicine, Nis, Serbia

**Keywords:** cancer stem cell, ovarian epithelial cancer, signaling pathways, pathogenesis, chemoresistance, therapy

## Abstract

The last decade witnessed an explosion of interest in cancer stem cells (CSCs). The realization of epithelial ovarian cancer (EOC) as a CSC-related disease has the potential to change approaches in the treatment of this devastating disease dramatically. The etiology and early events in the progression of these carcinomas are among the least understood of all major human malignancies. Compared to the CSCs of other cancer types, the identification and study of EOC stem cells (EOCSCs) is rather difficult due to several major obstacles: the heterogeneity of tumors comprising EOCs, unknown cells of origin, and lack of knowledge considering the normal ovarian stem cells. This poses a major challenge for urgent development in this research field. This review summarizes and evaluates the current evidence for the existence of candidate normal ovarian epithelial stem cells as well as EOCSCs, emphasizing the requirement for a more definitive laboratory approach for the isolation, identification, and enrichment of EOCSCs. The present review also revisits the ongoing debate regarding other cells and tissues of origin of EOCs, and discusses early events in the pathogenesis of this disease. Finally, this review discusses the signaling pathways that are important regulators of candidate EOCSC maintenance and function, their potential role in the distinct pathogenesis of different EOC subtypes, as well as potential mechanisms and clinical relevance of EOCSC involvement in drug resistance.

## INTRODUCTION

Despite its relatively low incidence rate, ovarian cancer is the most lethal type of gynecological malignancy and the fifth leading cause of cancer deaths among women[1,2]. Its high mortality rate is primarily due to the difficulties in diagnosis, including the lack of specific and sensitive biomarkers that could aid early diagnosis. The early-stage disease is commonly asymptomatic or presented with vague nonspecific symptoms. Most patients present with advanced-stage (III/IV) tumors, with an extremely low survival rate, vindicating its clinical nickname “the silent killer”. Furthermore, the early events leading to ovarian carcinoma development are poorly understood, thus complicating efforts to develop screening modalities for this disease[1,2].

Ovarian cancer represents a heterogeneous group of distinct tumors exhibiting a wide range of morphological characteristics, clinical manifestations, genetic alterations, and tumor behaviors. Based on the cell of origin, these tumors could be classified into three major types: germ cell, sex cord stromal, and epithelial. The malignant germ cell tumors arise from primitive germ cells in the ovary and account for around 5% of all ovarian malignancies. The sex cord stromal neoplasms are derived from mesenchymal cells in the ovarian cortex and represent <10% of all ovarian tumors[1,3]. Epithelial ovarian tumors represent the vast majority of all ovarian malignancies[1,2,4]. Epithelial ovarian cancers (EOCs) also represent a heterogeneous group of neoplasms and consist of various pathohistologic subtypes, as well as the diverse clinical manifestations, and underlying genetic and epigenetic alterations. Current clinical guidelines set forth by the World Health Organization (WHO) recognize eight histological EOC subtypes: serous, endometrioid, mucinous, clear cell, transitional cell, squamous cell, mixed epithelial, and undifferentiated. The three most common types – serous, endometrioid, and mucinous – are characterized by their morphological resemblance to various mucosal tissues of the female reproductive tract, as their name suggests[5]. However, the early events leading to EOC development are poorly understood, thus complicating efforts to develop screening or early detection modalities for this disease. Progress in understanding the biology of epithelial ovarian tumors is further complicated by the fact that their exact tissue of origin is still elusive. For decades, it was commonly believed that EOCs arise from the ovarian surface epithelium (OSE)**.** Recently, based on morphological, embryological, and molecular biological characteristics, it was suggested that they could arise from the extraovarian tissues with Müllerian origin[6,7]. Despite the advances in surgery and chemotherapy, the survival rate of patients with EOC remains extremely low. Aggressive surgical cytoreduction, followed by chemotherapy, results in clinical response in 50–80% of the patients with stage III and IV disease. Furthermore, the majority of patients will relapse and even become drug resistant[2,4]. This paper will therefore focus exclusively on EOCs, which constitute the predominant and most lethal forms of the disease.

The last decade witnessed an explosion of interest in cancer stem cells (CSCs). CSCs have been defined as a small subpopulation of cells within the tumor bulk mass that possess the capacity to self-renew and give rise to all heterogeneous cancer cell lineages that comprise the tumor of origin[8]. Therefore, the CSC population may generate tumors through the stem cell–like processes of self-renewal and differentiation into multiple cell types. Following the studies of leukemias, major advances have been made in this field with isolation, identification, and molecular studies of CSCs in solid tumors, such as those from the breast, brain, prostate, urinary bladder, pancreas, colon, and skin, establishing experimental proof of the CSC hypothesis in some tumors[9,10,11,12,13,14,15,16]. On the other hand, epidemiological studies identified multiple exogenous and endogenous risk factors for EOC development. The "two pathway model", based upon multiple oncogenic hits and rising genotoxicity, was recently proposed by Shih and Kurman in an attempt to integrate growing clinical, translational, and genetic evidence that support at least two broad categories of EOCs, which will be described in detail later[17,18,19,20]. However, the CSC hypothesis provides an attractive cellular mechanism to explain the therapeutic refractoriness, dormant behavior, and relapse of the disease, which poses a major therapeutic challenge in the case of EOC.

At present, all of the phenotypically diverse cancer cells in EOCs are treated as though all tumor cells have unlimited proliferative potential and can acquire the ability to metastasize. The concept that cancer growth recapitulates "stem-based" proliferative and/or regenerative processes, even though in very dysfunctional ways, has tremendous implications for cancer therapy. EOC represents a compelling and appropriate cancer that likely involves CSCs. The relative quiescence of the candidate EOC stem cell (EOCSC) subpopulation, resembling other stem cells, could spare them from cytotoxic effects of chemotherapy and allow them to initiate tumor recurrence, disease progression, and the development of drug resistance.

One of the broadly accepted methods for the isolation and study of CSCs is the clear correlation of the surface markers of specific stem/progenitor subsets and their malignant counterparts in the corresponding tumors. These include studies carried out in a variety of tumors, such as breast, brain, urothelial, prostate, and pancreas cancer[9,10,11,12,13]. Unfortunately, applying this approach to the EOCs where there is clearly a lack of information regarding the stem cell population in normal ovarian tissue is controversial, at the least. Lack of precise information regarding surface markers, expression patterns, and immunophenotyping in their normal counterparts poses a major challenge in the field of ovarian CSC research.

This review summarizes and evaluates the current evidence for the existence of both normal adult ovarian epithelial stem cells, as well as EOCSCs, emphasizing the requirement for a more definitive laboratory approach for isolation, identification, and enrichment of EOCSCs. This article also revisits the debate regarding other cells and tissues of origin of EOCs, and discusses early events in the pathogenesis of this disease. This review discusses in detail the signaling pathways that are important regulators of candidate EOCSC maintenance and function, their potential role in the distinct pathogenesis of different EOC subtypes, as well as potential mechanisms and clinical relevance of EOCSC involvement in drug resistance.

## ADULT OVARIAN SURFACE EPITHELIUM STEM CELLS

Adult stem cells have been identified and profoundly studied in most of the organs undergoing rapid cellular turnover[21,22,23,24,25,26,27]. However, despite the fact that female reproductive tract tissues undergo major remodeling events, as the physiological part of the reproductive cycle, adult stem cells in the ovaries seem to have been overlooked and understudied for a long time. Of the three primary functional somatic cell types of the ovary, the OSE and the theca cells currently have the most evidence supporting stem/progenitor cell biology. However, an understanding of the significant features of normal ovarian development is necessary before delving into stem cell functions of its particular somatic compartment. The development of the somatic gonad begins on E10 in the mouse (4 weeks gestation in the human) as a thickening of the coelomic epithelium on the ventromedial side of the mesoderm, the gonadal ridge, while the Müllerian ducts develop dorsolaterally from the urogonadal ridges[28,29,30,31]. The indifferent gonad is essentially a mass of blastema (the primordial mesenchymal cell mass) surrounded by the coelomic epithelium–derived surface epithelium. The epithelium proliferates and forms cords that penetrate into the ovarian cortex and give rise to the granulose cells of the primordial follicles. The follicles further become separated from the overlying surface epithelium by stromal cells[30,31].

Folliculogenesis in the adult ovary is characterized by extensive architectural remodeling that culminates in the disruption of the epithelial surface lining of the ovaries, the OSE, which was previously believed to give rise to new germ cells, therefore named the “germinal epithelium”, and the extrusion of the ovum at ovulation. The OSE is a single layer of cells continuous with the mesothelium of the ovarian ligament and the peritoneum[32,33]. Although the OSE represents only a small fraction of the wide variety of cells that populate the ovary, it is commonly accepted that a vast majority of malignancies attributed to this organ arise from OSE transformation[32,33,34,35].

However, there is still an ongoing debate as to whether all ovarian tumors actually arise from the ovary and it was proposed that they could instead arise from tissues that are embryologically derived from the Müllerian ducts, which will be discussed in detail in the next section[6,36]. The etiology and early events in the progression of these carcinomas are among the least understood of all major human malignancies, not only due to the complexity of the disease and organ of origin itself, but also due to major methodology obstacles in the laboratory research approach. Most of the studies regarding key functional and stem signaling pathways in the normal ovaries, such as Sonic Hedhog, Notch, Polycomb Wnt, and PTEN/PIK3, are still ambiguous and restricted to *Drosophila*[37,38,39,40,41] and mouse[42,43] models, which poses a major obstacle to the cancer stem signaling research**.** Furthermore, in the case of normal OSE research, there are still no appropriate animal models, while the methods to culture OSE have become available only recently[44]. To further complicate the matter, developed *in vitro* models are also limited by the life span of OSE cells in culture conditions in which their epithelial phenotype is maintained[44]. It should be stressed that many publications purporting to be evidence of certain cellular behavior are, in fact, derived from cell lines (from either human or high-quality mouse models) and therefore may not reflect unmanipulated cells.

After OSE disruption, a series of complex molecular events initiates and executes the repair of the epithelial wounds that is still not completely understood. This process of repeated disruption and repair during a long period of life, accompanied by complex remodeling, suggests a somatic stem/progenitor cell–mediated process. Furthermore, this might represent physiologically injury-responsive stem cells and their niches[45], which have been described in several tissues, such as skin and hair follicles[46,47], mammary glands[48,49], and the intestines[25].

Over the last few years, several studies have focused on the isolation, identification, and characterization of stem cells from the normal ovarian tissue[50,51,52,53,54], including those from the OSE. Recent studies have reported the isolation of cells from the OSE of postmenopausal women and women with premature ovarian failure that express developmental embryonic markers, including Oct4, VASA, Nanog, c-Kit, and Sox-2[50]. Using BrdU incorporation and doxycycline-inducible histone2B-green fluorescent protein pulse-chase techniques, Szotek et al. identified a label-retaining cell population in the OSE of the adult mouse ovary as candidate somatic stem/progenitor cells. The identified population showed quiescence, asymmetric label retention, a proliferation response to estrous cycling, significantly enhanced growth characteristics by the colony formation *in vitro*, and cytoprotective mechanisms[51]. OSE label-retaining cells were further characterized by the expression of the epithelial markers (cytokeratin-8 and E-cadherin) and the mesenchymal marker, vimentin, and enrichment in the cytoprotective ABC transporter-associated Hoescht dye-excluding side population (SP)[51], which has been associated with stem/progenitor cells in a variety of tissues and malignancies[55,56,57]. In addition, the fact that OSE cells exhibit a high degree of plasticity required for facilitating tissue remodeling (they express both epithelial and mesenchymal markers) and can transition from the epithelial to the mesenchymal phenotype has been well established[44,58,59,60,61]. However, the question of ovarian stem cell localization, characteristics, and differentional capacity is much more complex. The subpopulation of the potential stem cells derived from tunica albuginea showed the potency to differentiate into granulose cells, germ cells, and OSE[52,62]. On the other hand, granulose cells may revert toward the stem state, acquiring proliferation ability, mesenchymal markers, such as Oct4, and the capacity to differentiate into distinct lineages[54]. Thus, it has become imperative to further investigate normal adult ovarian stem cells in order to elucidate their potential role in ovarian cancer onset.

## EPITHELIAL OVARIAN CANCER PATHOGENESIS AND CANCER STEM CELLS

Substantial advances have been made to understand genetic alterations and molecular processes underlying EOCs. However the etiology remains poorly understood. The traditional view of EOC etiology assumes the accumulation of DNA damage by OSE cells, which become more susceptible to transformation, as well as the development of invaginations of these cells into the superficial ovarian cortex, which commonly become entrapped inside the stroma forming cortical inclusion cysts (CICs). In the new environment, the epithelium lining CICs is suddenly exposed to a completely new milieu of high concentrations of hormones, cytokines, and growth factors, which can initiate a differentiation or “metaplasia” and, eventually, a transformation process[30,63,64,65]. Physiologically, the OSE and the presumed stem and/or progenitor cells are subjected to ovulation-associated inflammation and reparation, various cytokines, growth factors, and reactive oxygen species that are capable of damaging DNA[64,66]. These damages may accumulate in the quiescent and slow-cycling stem and/or progenitor cells, and if these cells primarily bear some other genetic predispositions, they may become the prime targets for neoplastic transformation inside the CICs. The observation that women with a greater number of ovulatory cycles and aberrant ovulation have an increased risk of ovarian cancer supports the CIC hypothesis. *In vitro* models involving rat and mouse OSE cells in culture demonstrated transformation and chromosomal abnormalities with repeated passaging[32,67,68]. However, there are major weaknesses in this approach. This model neither explains the clear differences in genetic alterations that exist between tumor subtypes nor addresses differences in their outcome[7]. Furthermore, recent pathologic examinations of consecutive cases of ovarian primary peritoneal and fallopian tube cancers suggested that a greater percentage of ovarian cancers may actually have a fallopian origin with metastases to the ovary. Thus, it has been proposed that serous tumors arise from the implantation of the epithelium (benign or malignant) from the fallopian tube[7,69,70,71,72]. This sheds new light on the potential site of EOC origin, and therefore raises important questions regarding the characteristics of normal cells of origin and the initial processes in EOC development. Answering these questions requires novel experimental models of EOCs, as well as profound studying of other candidate somatic and/or stem/progenitor cells from the extraovarian tissue.

Some of the greatest challenges in the EOC research field stem from its heterogeneous nature. The “two pathway model” was recently proposed by Shih and Kurman in an attempt to integrate growing clinical, translational, and genetic evidence that support at least two broad categories of EOCs[17]. According to this model, type I tumors include all major histotypes (serous, endometrioid, mucinous, clear cell, and transitional) that exhibit low-grade nuclear and architectural features, exhibit slow growth, arise in a stepwise manner from borderline tumors[17], lack p53 mutations, and are relatively genetically stable[18,19]. The most common genetic alterations found among these tumors were KRAS and BRAF mutations, both of which are directly connected to the oncogenic MAPK signaling pathway[7,73,74]. The majority of genes altered in type I tumors are closely related to the process of epithelial-to-mesenchymal transformation (EMT)[75,76,77]. Through EMT, transformed epithelial cells can acquire the mesenchymal qualities that were confirmed to facilitate metastasis and a poor clinical outcome. The reactivation of the EMT program may result from either stable genetic alterations or through the exposure of cancer cells along the invasive front to factors present in the surrounding microenvironment[78,79]. More recently, the EMT program was shown to endow normal and transformed mammary epithelial cells with stem cell properties, including the ability to self-renew and efficiently initiate tumors[80,81]. This link between EMT and stem cells may have numerous implications in the understanding of the progression of EOCs. The EMT process may facilitate the generation of CSCs with the mesenchymal characteristics needed for dissemination, as well as the self-renewal properties needed for the initiation of secondary tumors. In contrast, another group of tumors, designated type II, for which morphologically recognizable precursor lesions have not been identified, is highly aggressive, evolves rapidly, and almost always presents in advanced stage. Type II tumors include conventional high-grade serous carcinoma, undifferentiated carcinoma, and malignant mixed mesodermal tumors (carcinosarcoma). It is now well established that they display TP53 mutations[18,19,20,82], may exhibit the overexpression of HER2/neu and AKT2[17,18,76], or, in the case of familial serous EOCs, BRCA1, BRCA2, MLH1, or MSH2[83,84,85,86]. All five genes function in DNA damage signaling and repair, suggesting that DNA damage is an especially important factor in the etiology of serous ovarian carcinoma[7,83,86]. However, this model also fails to answer the critical questions as to how type II tumors arise and what are the cells of origin.

Several studies utilizing high throughoutput genetic and other biomarker molecular profiling managed to identify “molecular signatures” that correlated with clinical outcome and predominantly with the “two-pathway model”, but there were also some surprises[71,72,87,88,89,90,91,92,93,94]. For example, the gene expression profile of clear cell tumors mostly correlated with clear cell tumors of other organs, such as renal clear cell carcinomas[90,92]. A microarray gene expression study of 285 serous and endometrioid tumors of the ovary, peritoneum, and fallopian tube showed that both endometrioid and serous tumors can exibit a large degree of molecular heterogeneity, clustering into six optimal subtypes. Two identified subtypes represented an analogue of the Shih and Kurman type I tumors (serous tumors with low malignant potential and low-grade endometrioid tumors). The remaining four subtypes represented higher-grade and advanced-stage cancers, which almost exclusively comprised C1 (high stromal response), C2 (high immune signature), C4 (low stromal response), and C5 (mesenchymal, low immune signature) subtypes, each associated with specific molecular and histopathology characteristics, as well as patient survival. A poor prognosis subtype was defined by a reactive stroma gene expression signature, which correlated with extensive desmoplasia[93]. Bearing in mind the results from CSC studies of other solid cancers, EOCSCs might also arise as a result of multiple various processes, such as the fusion of two cell types, dedifferentiation of committed progenitors, or even more differentiated cell types[11,95,96,97,98,99,100,101]. Therefore, one should not only consider the various cells of origin, but also the various molecular mechanisms leading to distinct EOCSCs in different EOC subtypes**.** These novel approaches should lead to a complete review of the EOC pathogenesis in light of the CSC hypothesis, opening new important pathways for this research field.

Furthermore, it seems that a greater percentage of ovarian cancers may actually have a fallopian origin with metastases to the ovary[7,69,70,71,72]. Inherited mutations in BRCA1 or 2 are associated with familial ovarian and breast cancer syndromes and account for ~11–15% of ovarian carcinomas, recommending women to undergo risk-reducing salpingo-oophorectomy; afterwards, precancerous lesions and early cancer lesions could be detected[7,102]. Studying fallopian tubes and ovaries of the BRCA+ patients, a few research groups reported dysplastic regions of the fallopian tube epithelium (which exhibited shift towards secretory phenotype, with complete loss of ciliated cells and positive Ki67 immunoreactivity) and/or occult tubal cancers, with no lesions present on the ovaries[7,69,71,103]. More recently, two profound studies reported a high incidence of serous tubal intraepithelial carcinomas in the fallopian tube, which were predominantly located in the fimbriated ends, with no lesions in the ovaries[72**,**104], suggesting that transformed fallopian tube epithelial cells may shed and subsequently implant into the ovaries or directly to the peritoneum[9]. Furthermore, it was shown that dysplastic lesions in the fallopian tube epithelium, fimbrias of BRCA+ women and control subjects, showed strong p53 immunoreactivity – “p53 signatures” associated with DNA damage marker γ-H2AX – providing the evidence that even under normal physiological conditions, fimbrial epithelial cells may undergo genotoxic damage[69,72,105,106]. It is speculated that inflammatory cytokines and reactive oxygen species associated with ovulation might be responsible for genotoxic stress in the fimbrial microenvironment[7]. Since fimbriae are in direct contact with the ovarian surface during ovulation, fimbriae also frequently adhere as a result of inflammation[6]. In light of these results, a model of ovarian cancer that proposes the fimbria as a major site of origin for serous carcinomas was developed. This model also includes two distinct molecular pathways similar to those involved in the formation of the Shih and Kurman type I and II tumors[105,107,108].

Dubeau[6] supported an extraovarian origin of EOCs and further proposed that primary ovarian epithelial tumors, fallopian tube carcinomas, and primary peritoneal carcinomas are all Müllerian in origin and nature, and could therefore be regarded as a single disease entity. The Müllerian (paramesonephric) ducts first develop as a pair medial to the mesonephric ducts early during fetal female development. Secretion of the Müllerian inhibiting substance by the testes prevents formation of the similar counterparts in males, which could provide an explanation for the lack of the analogical disease in males. The two Müllerian ducts eventually fuse in their distal portion to become the upper third of the vagina, cervix, and body of the uterus. The proximal segments remain unfused and become the fallopian tubes. The lower two-thirds of the vagina develops from an invagination of the skin that eventually connects to the Müllerian ducts[6,109,110]. It is well documented that EOCs are remarkably similar to epithelial cells from extraovarian sites in the female reproductive tract. Serous tumors morphologically resemble fallopian tube epithelium, endometrioid tumors resemble endometrioid glands, and mucinous tumors resemble the endocervical epithelium, all of which exhibit Müllerian differentiation and are notably unrelated to any structure of ovary[6]. On the other hand, malignant tumors that present as histologically and clinically identical to EOCs can be seen outside the ovary and can develop in patients that have undergone bilateral oophorectomy several years previously and for reasons other than cancer[111,112,113]. In addition, women with familial ovarian carcinoma predisposition owing to germline BRCA mutations continue to be at an increased risk of developing serous extraovarian carcinomas (usually referred to as primary peritoneal carcinomas) after undergoing prophylactic salpingo-oophorectomies[114,115,116]. Furthermore, Cheng et al. showed that serous, endometrioid, and mucinous ovarian carcinomas expressed the same set of HOX genes as epithelial cells from the normal fallopian tube, endometrium, and endocervix[117]. Although a substantial proportion of EOCs currently regarded as of primary ovarian origin seem to arise in the fimbriated end of the fallopian tube, this site cannot account for all of these tumors, even those with serous (fallopian tube–like) characteristics. It was proposed that these EOCs could be derived from components of the secondary Müllerian system[6].

Microscopic structures lined by the Müllerian epithelium grouped under the name "secondary Müllerian system" are very common in the paratubal and paraovarian areas, and they could also frequently be found inside the ovarian medulla and sometimes within the deeper portions of the ovarian cortex[118]. They also include endosalpingiosis, endometriosis, and endocervicosis. Thus, the various components of the secondary Müllerian system most likely possess the ability to provide a source for all the cell types that are present in the major subtypes of tubo-ovarian epithelial tumors[6]. This sheds new light on the potential site of EOC origin and raises important questions regarding the characteristics of normal cells of origin and the initial processes in EOC development. Answering these questions requires novel experimental models of EOCs, as well as profound studying of other candidate somatic and/or stem/progenitor cells from the extraovarian tissue.

Endometrial adult stem cells were recently isolated and showed the surprising ability to differentiate into cell types of all three germ layers[119,120,121,122,123,124]. These studies, while valuable, require further profound analysis in order to validate proposed molecular markers and much more important functional characteristics of the endometrial adult stem cells. However, this novel adult stem cell population could provide a promising model for the investigating pathogenesis of endometriosis and an endometrial subtype of EOCs. Jazedje et al. recently managed to isolate, expand, characterize, and access the differentiation potential of adult mesenchymal stem cells from human fallopian tubes. The presumed stem population showed high levels of adhesion markers (such as CD29, CD44, and CD90) and mesenchymal stem markers (such as CD13, CD73, SH2, SH3, and SH4), were HLAI positive, and showed an ability to differentiate into muscle, fat, cartilage, and bone tissue *in vitro*[125]. There are no publications regarding the existence of the stem cells in the columnar epithelium of the fallopian tubes in the present literature. On the other hand, a recent gene expression profiling study revealed the multipotent ability of OSE stem cells, which supports the hypothesis that they could be capable of serving as EOC initiating cells[126]. The C5 cancers proposed by Tothill et al. shared some characteristics with normal OSE, including overexpression of N- and P-cadherin, reduced expression of E-cadherin, and low expression of differentiation markers, such as CA125, Muc1, and Muc16[93]. It seems most likely that both ovarian and extraovarian tissues may contribute to EOC development, and that the potential OSE stem origin of at least some of these tumors should not be ruled out.

Research of normal stem cells in the female reproductive tract is still in its infancy and certainly should be encouraged. This knowledge could be essential to understand the mechanisms underlying the risk factors and early events in EOC pathogenesis, and could be crucial for the development of effective screening protocols aimed at its early detection.

## EPITHELIAL OVARIAN CANCER STEM CELLS

The realization of EOC as an aberrant stem cell disease has the potential to change approaches to diagnosis and treatment dramatically[127]. However, compared to the CSCs of other cancer types, the identification and study of EOCSCs is much more difficult due to several major obstacles: the heterogeneity of tumors comprising EOCs, the controversial results and undefined etiology and pathogenesis of EOCs, the unknown origin, and the normal adult stem cell population not being definitely identified and analyzed. This poses a major challenge for urgent development in this research field.

The first study on the isolation and identification of candidate EOCSCs was reported just a few years ago[128]. Bapat et al. presented evidence that the aggressiveness of the human ovarian cancer may result from the dysfunctional stem-like population. An *in vitro* model comprising 19 spontaneously immortalized clones isolated from various malignant cells derived from the ascites of a patient with advanced serous ovarian adenocarcinoma showed a single tumorigenic clone. During the course of this study, another clone underwent spontaneous transformation in the culture, gaining tumor-forming ability. Both clones were shown to possess stem cell–like characteristics and the ability to grow in an anchorage-independent manner *in vitro* (as spheroids), and to form multicellular colonies in soft agar, although further maturation and tissue-specific differentiation was arrested. All xenograft tumors established from these two clones in animal models showed similar histopathology and cell architecture to those in the human cancers. Furthermore, these particular clones continued to establish tumors even on serial transplantation, thereby presenting functional evidence of the association of cancer cells with stem-like features with serous ovarian adenocarcinoma. Surprisingly, all 19 clones expressed some stem-related markers, such as c-Kit (CD117), stem cell factor (SCF), CD44, as well as EGFR and E-cadherin[128]. However, several vigorous studies with more lines developed from different patients, as well as different types of EOCs, are still needed to prove indisputably the existence and nature of EOCSCs.

CSC isolation from tumors classically exploits the detection of cell surface markers associated with normal stem cells. However, discrepancies in such approaches have been noted, especially because quiescence has not been shown in some cells positive for these markers[129]. Bapat recently profoundly reviewed the value of current candidate EOCSC surface markers, such as CD133, CD44, c-Kit (CD177), and MYD88, discussing the significance, possible function, as well as some controversies regarding different single- and double-positive subpopulations isolated from ovarian cancers[127]. RT-PCR analyses revealed distinct microRNA (miRNA) expression profiles between CD133+ and CD133- OVCAR3 cells. The expression of stem cell–specific genes, namely, Oct3/4, Sox-2, and Nanog, was higher in CD133+ cells than in CD133- cells, while that of FoxD3 was lower in CD133+ cells than in CD133- cells[130]. In an attempt to understand and overcome major clinical barriers in the diagnosis and therapy of EOCs, it may be of particular interest to subject candidate EOCSCs to a functional assessment, such as quiescence and/or drug-resistance capabilities, rather than the surface phenotype.

In addition, the specific phenotype exhibited in the candidate EOCSCs is a result of many overlapping genetic, epigenetic, transcriptional, and translational regulation events. A recent study revealed that CD133 transcription is controlled by both histone modifications and promoter methylation. Sorted CD133- ovarian cancer cells treated with DNA methyltransferase and histone deacetylase inhibitors showed a synergistic increase in cell surface CD133 expression. In addition, DNA methylation at the P2 promoter, which is active in ovaries, inversely correlated with CD133 transcription. Furthermore, promoter methylation increased CD133- progeny of CD133+ cells, and CD133+ cells preserved a less methylated or completely unmethylated state[131]. A recent transcriptional study proposed that EOCSCs coexpress Lin28 and Oct4, based on the analyses of both cell lines and patient tumor samples. Combined expression of these proteins in tumor samples correlated with advanced tumor grade, whereas the repressed expression of these two proteins using RNA interference showed significant reduction in cell growth and survival. Lin28 and Oct4 are highly expressed in human embryonic stem cells. As an RNA-binding protein, Lin28 acts to stimulate the translation of a specific subset of mRNAs, and along with Oct4 transcription factor, plays a key role essential for the maintenance of pluripotency and survival of embryonic stem cells. Thus, Lin28 and Oct4 may have important roles in the initiation and/or progression of EOC and, consequently, may serve as important molecular diagnostics and/or therapeutic targets for the development of novel treatment strategies in EOC patients[132].

"Side population" (SP) cells could be defined as a small subset of cells with a property of active discard of certain molecules taken up by cells (such as the dye Hoechst 33342). Therefore, SP cells can be efficiently isolated by flow cytometry sorting. The SP method application has drawn special attention to the field of stem cell research and many studies utilize this method to enrich candidate stem cells in many normal and malignant tissues[56,133,134,135,136,137,138,139]. SP cells isolated from EOCs share many properties of presumed EOCSCs. Using Hoechst 33342 dye efflux, Szotek et al. identified and characterized SP cells from two distinct genetically engineered mouse ovarian cancer cell lines (MOVCAR7 and 4306) with the capacity for self-renewal and production of heterologous non-SP progeny[56]. In *in vivo* assays, SP cells showed a higher tumor-forming ability. The Müllerian inhibiting substance inhibited the proliferation of both SP and non-SP cells in contrast to the lipophilic chemotherapeutic agents, such as doxorubicin, where SP showed significant chemoresistance. SP was also identified in the human ovarian cancer cell lines IGROV-1, SKOV3, and OVCAR3, and in cells from patient ascites, although in a much smaller number[56].

Recently, Gao et al. reported a detailed study aimed at delineating optimization parameters required to identify and isolate SP cells in the ovarian cancer cell line OVCAR3. Results showed that SP

accounted for about 0.9% of the whole cell population. The sorted SP cells showed significantly higher colony formation efficiency than the non-SP cells, and only the SP cells could form holoclones, had enriched tumor-forming capacity, and were resistant to chemotherapeutic drugs. RT-PCR disclosed that SP cells expressed significantly higher levels of the "stemness"-related gene Oct3/4, indicating that the SP cells might harbor ovarian cancer CSCs. This report emphasized a need for SP isolation optimization in cell studies whenever these populations are to be considered[137]. SP cells isolated from ascites derived from EOC patients and from mice inoculated with human ovarian cancer cell lines expressed stem-related cell markers, such as Oct4, Nanog, STELLAR, and ABCG2/BCRP1[140].

The presence and significance of SP cells in tumor cell lines is still being debated, but differences might be due to the requirements of different technical modifications, especially when cell staining and heterogeneity of EOC cell cultures is considered[137]. However, Moserle et al. investigated the presence of SP in EOCs and xenotransplants, and confirmed it in only nine of 27 primary tumor samples, as well as in four of six cultures from xenotransplants[141]. SP cells again showed higher proliferation rates, apoptotic resistance, and significant tumorigenic ability compared to non-SP cells. IFN-α, a cytokine that has widely been used to treat solid tumors, exerted significant antiproliferative and proapoptotic effects on primary cultures containing high numbers of SP cells, and was related to a distinctive change in their transcriptional profile, which was not observed when tumors bearing low SP levels were treated[141]. These findings could have major clinical implications for EOC treatment if the particular tumor possesses a confirmed significant SP.

Long-term label-retention assays that rely on quiescence and the slow-cycling nature of stem cells were used in several studies to define and/or enrich the candidate CSC population in various tumors[142,143,144,145,146,147,148]. Because label quenching is a direct function of fluorochrome dilution following each consecutive cell division, the profiles generated could strongly correlate with the subsistence of proliferative heterogeneity within primary tumors and their metastases. Although BRDU labeling of DNA is the most common technique, a recent study showed its cytotoxic nature, raising the question of more applicable assays[149]. Recently, Kusumbe et al.[148] used vital membrane-labeling dyes PKH67/PKH26, which showed similar results as BRDU assays[150,151], to identify a quiescent cell subpopulation in the A4 cell line established from malignant ascites from a patient with high-grade serous ovarian adenocarcinoma[128], as well as commercial human tumor cell lines NT2, PA1, HL60, C6, U87, and T47D. The authors proposed that EOCs consist of three distinct populations: (1) label-retaining $PKH^{hi}$ cells, suggested to be slow-cycling/quiescent – the candidate EOCSCs; (2) $PKH^{lo}$ cells that undergo partial label dilution indicative of limited divisions – the candidate tumor progenitor cells; and (3) $PKH^{neg}$ cells that undergo total dye quenching suggestive of consecutive, rapid divisions – "differentiated" tumor bulk cells[148]. Metastases-derived cells also showed three presented fractions. $PKH^{hi}$ cells showed CSC characteristics, such as self-renewal, high tumor-forming ability in xenograft assays, and the expression of stem-related markers Oct4, Nestin, Nanog, Bmi, CD44, and c-Kit. The identification of EOCSCs as label-retaining $PKH^{hi}$ cells that undergo reversible quiescence through functional assay of clonogenicity *in vitro* and tumorigenicity *in vivo* provided the first indication of their involvement in tumor dormancy[148]. Furthermore, the survival and persistence of their functionality during therapeutic regimes would provide a defining validation in EOCs[127]. PKH labeling enables the monitoring of live cells, which could be of significant value in the enrichment of EOCSCs in *in vitro* studies of primary tumors, metastases, as well as EOC commercial cell lines. However, more profound studies of stem-like cell lines developed from a significant number of patients with various forms of EOC are urgently needed to profoundly evaluate all these experimental approaches.

From a clinical perspective, all these findings illustrating the contribution of EOCSCs to the development and evolution of disease could have significant therapeutic implications towards the development of a new generation of EOC therapies specifically targeting the EOCSC population. However, this also poses a major challenge to urgently develop more reliable and accordant methodology for the identification and isolation of putative EOCSCs.

## OVARIAN CANCER STEM CELLS SIGNALING PATHWAYS

Although there has been considerable progress in cancer stem cell research in a variety of tumor types, including EOC, the pathways that drive the homeostasis of these cells are not well understood. Currently, genetically engineered models of human cancer through the expression of oncogenes, specific genetic mutations, or the inactivation of tumor suppressor genes, and human cell lines (whether commercial or developed from patients tissue) that might also be genetically engineered, have begun to provide us with an understanding of the molecular pathways involved in CSC initiation, maintenance, and tumor progression at the functional level. The focus on targeted therapies has been fueled by extensive research on CSC molecular pathways and their role in tumorigenesis.

### The Canonical WNT/β-Catenin Pathway

The WNT signaling pathway is evolutionary, very conserved, and controls many essential biological processes modulating the delicate balance between the stem cell pool, differentiation, and proliferation in several adult stem niches. This signaling pathway is tightly regulated by diverse proteins and provides cellular polarity, regulation of the cell cycle, and cell adhesion during both the embryonic and the adult period[152,153].

While WNT signaling plays a key role in the normal embryonic development of the ovary, as well as normal follicular development and ovarian function[154,155,156,157], there is mounting evidence implicating abnormal WNT signaling in ovarian tumorigenesis[158,159,160,161,162]. However, it should be noted that reports written regarding β-catenin expression patterns in EOCs are sometimes conflicting. Two clinic-based case-control studies at the Mayo Clinic were assessed, including 798 breast cancer cases, 843 breast cancer controls, 391 ovarian cancer cases, and 458 ovarian cancer controls, which revealed no associations with the risk of breast or ovarian cancer[163]. Almost 2 decades ago, several authors began to detect the presence of β-catenin gene mutations in EOC, mostly confined to the endometrioid subtype[158,159,164]. Oncogenic missense mutations affect the NH2-terminal regulatory domain of β-catenin (codons 32−45), which overlaps with the consensus sites for GSK3β phosphorylation and ubiquitin-proteosome degradation. In addition, it was demonstrated that both mutations in the CTNNB1 gene and mutations in the genes that encode components of the degradation complex, such as the APC, AXIN1, and AXIN2 in the target cell cytosol, affect the uncontrolled activation of WNT signaling[164,165]. A presumed critical consequence of WNT pathway mutations, whether in the CTNNB1, APC, or AXIN genes, is the elevation of β-catenin levels in the cytoplasm and translocation to the nucleus, which results in activating the aberrant gene transcription, contributing to the initiation of the endometrioid type of EOC. The methylation of the genes that encode regulators of the WNT signaling cascade seems to have a critical role in endometrioid EOC formation[161,162,166]. Methylations in the SFRP1 gene promoter region repressed its expression and this silence resulted in the abnormal activation of WNT/β-catenin signaling via truncated sFRP protein, which is an important antagonist of WNT/β-catenin signaling[166]. The gene expression study in primary ovary endometrial carcinomas, with the intact vs. the deregulated WNT, showed a nearly fivefold increase in the expression of the fibroblast growth factor 9 (FGF-9) signaling in carcinomas with WNT pathway defects compared to tumors with intact WNT signaling. FGF-9, also known as the glial-activating factor, provides an experimental target for repressing hyperactive WNT signaling[167].

However, the nuclear expression of β-catenin was proposed as an indicator of good prognosis and complete loss of its expression was related to the poor prognosis[158,164,168], possibly linking WNT/β-catenin signaling to the Shih and Kurman type I EOCs. A recent retrospective study, which addressed the association of β-catenin expression in endometrioid EOCs and correlated it with the Gynecologic Oncology Group's grading system, clinicopathological characteristics, and patient survival, revealed low membranous expression of β-catenin and high mitotic count as poor prognostic indicators in patients with endometrioid EOCs[169]. The high-grade Tohill's C5 group of EOCs harbored an expression profile suggesting a mesenchymal or dedifferentiated phenotype. C5 EOCs showed overexpression of genes associated with WNT signaling, developmental transcription factors in combination with reduced membranous E-cadherin staining, which was suggestive of involvement of EMT in carcinogenesis[93]. Ovarian cancer epithelial cells (CEPI) isolated by laser capture microdisection from human serous papillary ovarian adenocarcionoma showed significantly lower levels of inhibitors of differentiation, WNT2B and WNT5A, compared to OSE. In contrast, WNT7A (the potent inducer of replication) was highly expressed in CEPI[126]. These results nominate WNT signaling as a promising therapeutic target for EOCSCs in the Shih and Kurman type I EOCs. Besides the possible effects of the WNT pathway on the initiation and progression of these tumors, it seems that the evolution of drug resistance is significantly modulated utilizing the up-regulation of the WNT pathway as an escape route. Shutting off such evasion maneuvers of resistant ovarian cancer cells may be pivotal to developing targeted therapies in ovarian cancer.

## Sonic/Hedhog Pathway

The Hedgehog (Hh) signaling pathway is vital for a variety of aspects of animal development and essential in humans during development[170,171,172]. Recent studies have also implicated Hh signaling as essential for the maintenance of stem cell homeostasis[173,174,175,176,177]. Vertebrate organisms possess three Hh proteins: Sonic Hh (Shh), Indiana Hh (Ihh), and Desert Hh (Dhh), which all bind to the same receptor, PATCHED1 (PTCH1). Without ligand stimulation, Ptch1 restrains signaling of the seven-pass transmembrane protein and proto-oncogene Smoothened (Smo). Upon Hh binding, this inhibition is relieved and Smo transduces the signal to the ultimate effectors of the pathway, the zinc-finger transcription factors Gli1, Gli2, and Gli3[178]. Hh signaling is abnormally activated in many types of tumors, including gliomas, gastrointestinal, prostate, pancreas, and breast cancers. In several tumors, such as gastric and prostate cancers, Hh signaling activation was associated with aggressive cancer progression. The activation of Hh signaling has been reported in about 30% of human cancers, including ovarian cancer[179,180,181,182,183,184,185,186,187]. The overexpression of Hh ligands is believed to be responsible for activated Hh signaling. Overexpression of Patched and Gli1 protein in ovarian cancers correlated with poor survival of the patients. Significantly elevated expression of Shh messenger RNA was observed in various subtypes of EOCs (serous, mucinous, endometrioid, and clear cell carcinomas) compared with normal tissues and benign ovarian tumors, and such differential expression was specific to histological types[186]. Surprisingly, the expression of Ptch1, a direct transcriptional target of the Hh pathway, was down-regulated in the EOC cell lines in direct contrast to the expression observed in other adult solid tumors. The expression of BMI-1, a polycomb gene, was also down-regulated in EOC cells following cyclopamine treatment. The overexpression of PTCH1 phenocopied the effects of cyclopamine; it down-regulated BMI-1 and reduced clonal growth in ovarian cancer cell lines[187]. It seems that the constitutive low-level expression of PTCH1 may contribute to the proliferation and clonal exclusion of EOC cells by an aberrant Hh signal. The modulation of Gli1 expression in the stromal compartment implies signaling between the tumor and the stroma. Ectopic Gli1 overexpression showed promise as an independent prognostic marker, and it correlated with increased cell proliferation, cell mobility, invasiveness, and change in differentiation in association with increased expression of E-cadherin, vimentin, Bcl-2, and caspases, as well as β1 integrin, membrane type 1 matrix metalloproteinase (MT1-MMP), and VEGF[186,187,188].

The inhibition of Hh signaling has been pursued as an effective strategy for cancer treatment[189], including an ongoing phase II clinical trial in EOCs[190,191]. The current challenges for therapeutic application of Hh signaling inhibitors include the identification of the right tumors for therapeutic application, reliable and reproducible animal models for testing these compounds, and the optimization of drug dosages to minimize the side effects.

However, one must bear in mind that the rate of Hh signaling activation in ovarian cancer was reported differently by different groups and the obtained results are sometimes conflicting[186,191,192,193]. One of the answers might lay in the very nature of Hh proteins. The Hh proteins Shh, Ihh, and Dhh are secreted molecules, functioning both on nearby and distant cells in developing tissues, and there is a significant possibility that this property is retained in adult tissues at some level. A hallmark of Hh signaling is its ability to act over a long range and control distinct cell fates as a function of Hh concentration[194], raising important questions of how Hh gradients are generated and maintained during development and in stem cell niches, and how different thresholds of Hh are transduced to elicit distinct outcomes. In addition to the signaling strength, signaling duration is also important for shaping developmental outcomes, raising questions of how the responses to Hh signaling change over time and how the signals are terminated. The next compelling question is the proven connection between tumors and the Hh and WNT pathways[179,195,196]. Answering what the level is, and what particular molecule mechanisms of two signaling cascades cooperation are, might contribute to understanding the process of initiation of the ovarian CSCs**.** It is also possible that the involvement of Hh signaling in human ovarian cancers may be context dependent, occurring in some tumor cell lines, but not in others. Evidence suggests that Hh signaling may be involved in maintaining ovarian CSC proliferation[190,197].

Nevertheless, more comprehensive study on ovarian cancer Hh signaling is necessary in order to predict the feasibility of clinical trials of Hh signaling inhibitors in ovarian cancer. Furthermore, the dubious data on the expression of Hh in ovarian cancer patients raise a question of identifying the right population of ovarian cancer patients in the clinical trials with Hh signaling inhibitors using powerful genomic and proteomic tools.

## The Notch Pathway

The Notch pathway, with its family of four mammalian Notch receptors and their numerous ligands of the Delta and the Jagged/Serrate group, is a developmental, conserved signaling pathway that plays a fundamental role in embryonic development and adulthood[198,199,200]. In developmental processes, Notch signaling often results in the preservation of a small subset of proliferating, undifferentiated, and multipotent progenitor cells. It also has a critical role in the self-renewal of the adult tissues, controlling *a priori* the differentiation of the stem cells[198,201,202,203,204]. It seems likely that the deranged proliferation and cell fate determination processes are of particular importance in the development of ovarian cancer. The *in vitro* study demonstrated that p73, a member of the p53 family, is expressed in ovarian adenocarcinoma, but rarely in adenoma, which would correlate with the Shih and Kurman type II tumors[19,205]. This poses Notch signaling as a potential molecular target for high-grade and aggressive EOCs. P73, p63, and other members of the p53 family were shown to directly increase the expression of the Notch ligands Jagged1 and 2, leading to the increased expression of the Notch target HES1 in coculture assays[206].

Notch3 expression was widely studied in high-grade EOCs[207,208,209]. The gene amplification seems to be one of the up-regulating mechanisms of Notch3. The analysis of ovarian high-grade serous carcinomas showed the amplicon at 19p13.12 in all 34 genes within the minimal amplicon-identified Notch3 as the gene that showed most significant overexpression in amplified tumors compared with nonamplified tumors. The Notch3 DNA copy number is positively correlated with Notch3 protein expression. The inactivation of Notch3 by both the γ-secretase inhibitor and the Notch3-specific small interfering RNA (siRNA) suppressed cell proliferation and induced apoptosis, but only in the cell lines that overexpressed Notch3[207]. Compared to low-grade tumors, high-grade serous carcinomas showed widespread DNA copy number changes. The most frequent alterations were in loci harboring candidate oncogenes: cyclin E1 (CCNE1), AKT2, Notch3, and PIK3CA, as well as in novel loci, including 12p13, 8q24, 12p13, and 12q15. A high level of DNA copy number gain was found in CCNE1, Notch3, HBXAP/Rsf-1, AKT2, PIK3CA, and chr12p13 occurring in 36.1, 7.8, 15.7, 13.6, 10.8, and 7.3% of high-grade serous carcinomas[209], respectively. The study of the mRNA and protein expression of Notch3, Jagged1, and Jagged2 in EOCs detected high levels of Notch3 mRNA and protein expression especially in serous ovarian carcinomas compared to their benign counterparts, accompanied by a positive correlation with the expressions of Jagged1 and 2. The Notch3 expression level was therefore proposed as a potentially independent poor prognostic factor in this subset of tumors[210].

It seems that Jagged1, the primary Notch3 ligand in ovarian carcinoma, and Jagged1/Notch3 interaction may constitute a juxtacrine loop, promoting proliferation and the dissemination of ovarian cancer cells within the intraperitoneal cavity. Jagged1 was shown as the highest expressed Notch ligand in the EOC cells, as well as in peritoneal mesothelial cells that are in direct contact with the disseminated EOC cells. The cell-cell adhesion and cellular proliferation were reduced in Notch3-expressing EOC cells that were cocultured with Jagged1 knockdown mesothelial and tumor feeder cells. The interaction of Notch3-expressing EOC cells with Jagged1-expressing feeder cells activated the promoter activity of candidate Notch3 target genes, and this activity was attenuated by Notch3 siRNA. Constitutive expression of the Notch3 intracellular domain significantly suppressed the Jagged1 siRNA–mediated growth inhibitory effect. In the Notch3-expressing EOC cells, Jagged1-stimulating peptides enhanced cellular proliferation, which was suppressed by γ-secretase inhibitor and Notch3 siRNA[211].

However, it seems that Notch1 is also significantly active in EOC carcinogenesis and that the level of activation might even correlate with the aggressive and resistant cellular phenotype. The expression of Notch1 pathway elements assessed by genetic and immunotechniques showed a significantly higher and more frequent expression of Jagged2, Delta-like-1, Manic Fringe, and TSL1 adenocarcinomas, whereas Deltex, Mastermind, and Radical Fringe were more frequent in adenomas. Quantitative PCR revealed decreased Notch1 mRNA in ovarian adenocarcinomas, compared with adenomas, again confirming the significance of Notch signaling in type II EOCs. However, HES1 protein was strongly expressed in 18/19 ovarian cancers and borderline tumors, but not in adenomas[212]. The study of the candidate ovarian cancer–initiating cells isolated from ovarian serous adenocarcinomas demonstrated an up-regulation of multiple stem cell regulators (Notch, Bmi-1, stem cell factor, Nanog, Nestin, ABCG2, and Oct4) compared with parental tumor cells. This up-regulation also correlated with enhanced chemoresistance to the ovarian cancer chemotherapeutics cisplatin or paclitaxel[213].

The active form of Notch1, the Notch1 intracellular domain (NICD), seems to be overexpressed in EOC cell lines and the depletion of NICD may therefore lead to growth reduction, providing a novel target for ovarian cancer therapy. NICD was significantly expressed in cell lines (OVCAR3, SKOV3, and CaOV3), but also human serous EOCs. Furthermore, the depletion of Notch1 led to the growth inhibition of EOC cells[214]. Notch1 and HES1 were found in the entire four human (A2780, SKOV3, HO-8910, and HO-8910PM) and one ovarian surface (IOSE 144) cell lines, and they were significantly higher in A2780 compared to the other four ovarian cells. The release of the NICD is mediated by γ-secretase. The γ-secretase inhibitor N-[N-(3,5-difluorophenacetyl)-l-alanyl]-S-phenylglycine t-butyl ester (DAPT) has been shown to be a potent inhibitor of NICD, and led the growth inhibition and apoptosis of A2780 cells in a dose- and time-dependent manner[215]. These data support that blocking the Notch1 activity, by γ-secretase inhibitors, for instance, represents a potentially attractive strategy for a targeted therapy for high-grade EOCs.

Therapeutic strategies based on antiangiogenic approaches are beginning to show great promise in clinical studies. Notch1 and 4 function in developing the endothelium, whereas Notch3 is critical for smooth muscle cell differentiation. Notch ligands involved in the process of angiogenesis include Delta-like-1, Delta-like-4, and Jagged1. Furthermore, Notch components were strongly expressed in tumor vessels, most notably Delta-like-4, compared to adjacent normal vessels[215,216,217,218]. The gene expression study of the differences in purified endothelial cells from 10 invasive EOCs and five normal ovaries, using the microarray technique, identified more than 400 differentially expressed genes in the tumor-associated endothelial cells. Among these, the Polycomb group protein enhancer of Zehste homologue 2 (EZH2), the Notch ligand Jagged1, and PTK2 were elevated 3- to 4.3-fold in tumor-associated endothelial cells. Silencing these genes individually with siRNA blocked endothelial cell migration and tube formation *in vitro*[219].

## PTEN/AKT Pathway

The PTEN/AKT pathway is implicated in signal transduction through tyrosine kinase receptors and heterotrimeric G protein–linked receptors. The PTEN is a tumor-suppressor gene whose mutations/deletions, as well as promoter methylation gene silencing, are found at a high frequency in many primary and metastatic human cancers[220,221,222,223]. PTEN inactivation is also associated with poor patient outcomes, aggressive tumor growth, and resistance to chemotherapy and current targeted therapies[223,224,225,226,227]. PTEN leads to AKT and downstream pathway down-regulation. Thus, the resistance to various apoptotic stimuli, the deregulation of self-renewal, and increased and/or abnormal cell growth have direct consequences on abnormal AKT activation alone, and due to the PTEN deficiency as well[228,229].

There is a complex network of associations with other CSC regulators and growth factor signaling pathways. It was documented that PTEN is directly associated with p53 increasing protein stability, protein levels, and transcriptional activity[230]. PTEN mutations are very common in tumors of the endometrioid type, and it was well documented that loss of heterozygosity (LOH) at locus 10q23.3 and mutation of the PTEN occur frequently in endometrial carcinoma[231] and ovarian endometrioid carcinoma, as well as endometrioid carcinoma synchronous with endometriosis[232]. In addition, the majority of tumors with PTEN mutations were grade 1 and/or stage 1[231,233]. In light of the “two pathway model”, aberrant PTEN signaling may be an early event in type I cancer development, along with KRAS, BRAF, and β-catenin alterations.

AKT is proposed as a determinant of capsulation (cis-diammine-dichloroplatinum [CDDP]) resistance in EOCs, which may be related to the up-regulation of p53. AKT is an important regulator of both X-linked IAP (XIAP) and p53 levels after CDDP challenge, and the functional p53 status is a determinant of AKT-mediated chemoresistance[234,235]. Precisely how AKT facilitates CDDP resistance and interacts with p53 is still unclear. However, recent evidence suggests that the failure of drug-induced apoptosis may be an underlying factor of chemoresistance. The second mitochondria-derived activator of caspases (Smac), also known as direct inhibitor of the apoptosis protein (IAP) binding protein with low isoelectric point (DIABLO), is released from mitochondria into the cytosol in response to apoptotic stimuli[236]. Smac release is regulated by p53 via the transactivation of proapoptotic Bcl-2 family members. The study of CDDP in EOC cell lines, both chemosensitive (OV2008 and A2780s) and chemoresistant (C13* and A2780cp), suggested that CDDP may induce mitochondrial p53 accumulation followed by the release of Smac, cytochrome c, and HTR/Omi, and apoptosis in chemosensitive, but not in resistant, EOC cells. AKT attenuated mitochondrial p53 accumulation and Smac/cytochrome c/Omi release, and conferred CDDP resistance. The inhibition of AKT facilitated Smac release and sensitized chemoresistant cells to CDDP in a p53-dependent manner[237]. Therefore, AKT is a promising molecular target for overcoming developing chemoresistance in the Shih and Kurman type I EOCs. AKT kinase inhibitors are still in development; however, as a first test of this hypothesis, phase I and II trials of inhibitors of mTOR, namely, rapamycin and rapamycin analogs, are under way. A recent study raised the possibility that drugs targeting other kinases, or PI3K itself, might have significant therapeutic activity in PTEN-null cancers[238].

## MicroRNAs

MicroRNAs (miRNAs) represent a novel class of molecules that function as negative regulators of gene expression. Recently, miRNAs have been implicated in several cancers. Yang et al. showed a frequent deregulation of *miR-214*, *miR-199a**, *miR-200a*, and *miR-100* in ovarian cancers. *miR-214* seems to be responsible for cell survival and cisplatin resistance through the targeting of the 3′-untranslated region (UTR) of the PTEN, which leads to the down-regulation of the PTEN protein and the activation of the AKT pathway. The same group using the AKT inhibitor, API-2/triciribine, or the introduction of PTEN cDNA lacking 3′-UTR largely abrogates *miR-214*–induced cell survival[239]. These findings indicate that the deregulation of miRNAs is a recurrent event in human ovarian cancer and that *miR-214* induces cell survival and cisplatin resistance primarily through targeting the PTEN/AKT pathway. A more recent study with EOCSCs (type I/CD44+) shows that type I/CD44+ cells were characterized by low levels of both *miR-199a* and *miR-214*, whereas mature EOC cells (type II/CD44−) had higher levels of *miR-199a* and *miR-214*. Furthermore, this study proposed Twist1 as a regulator of this unique miRNA cluster responsible for the regulation of the IKKβ/NF-κB and PTEN/AKT pathways, and its association of ovarian CSC differentiation. The regulation of *MIR199A2/214* expression may be used as a potential therapeutic approach in EOC patients. Twist1 may be an important regulator of "stemness" in EOC cells and, therefore, a promising target as well[240]. Molecular targeting of distinct oncogenic signaling elements related to "stemness" activated in the EOCs may be new therapeutic valuable strategies in future research.

## DRUG RESISTANCE AND EOCSCS

One of the greatest clinical challenges and the most important causes of failure in EOC treatment is the development of chemoresistance. A significant number of patients that initially respond to standard combinations of surgery and chemotherapy later develop a recurrent, therapy-resistant lethal disease[241,242].

In light of the CSC hypothesis, there is possibly a pool of EOCSCs behind the development of drug-resistant ovarian tumors. One of the reasons for the acquisition of chemoresistance could be their primary stem-like properties, such as quiescence, self-renewal, antiapoptotic, and other self-preserving mechanisms, allowing EOCSCs to survive the therapeutic regimen and later give rise to the new tumor. The identification of EOCSCs as label-retaining PKH$^{hi}$ cells that undergo reversible quiescence provided the first indication of their involvement in tumor dormancy. Both short- and long-term chemotherapy regimens with paclitaxel applied on PKH-labeled A4 cells augmented PKH$^{hi}$ cells in tumors, suggesting an active enrichment of EOCSCs, which was not the consequence of drug-mediated accumulation of label-retaining senescent or apoptotic cells[148]. Evidence of the survival of EOCSCs and the persistence of their functionality during other chemotherapeutic regimes would provide a prognostic validation of different forms of EOCs and propose novel clinical approaches. Furthermore, if the quiescence of CSCs operates like a safeguard mechanism, then the therapy-mediated selection and enrichment of resistant EOCSCs may be one of the important factors for the development of the clinically refractory disease, especially in repeated drug exposure regimens[127,148].

Besides harboring CSCs, most solid tumors contain aneuploid populations that represent genetically diverse cell pools with reduced cell proliferation and cellular fitness. In a small subset of such cells, however, cellular imbalances associated with increased mutation rate, gene amplification, and/or genomic instability have linked aneuploidy with tumor dormancy[243,244,245,246,247]. From this point of view, Kusumbe and Bapat recently proposed two parallel mechanisms of developing refectory disease: the stem-like potential of tumor-derived EOCSCs to survive, persist, and retain functionality through drug regimes; and acquisition of proliferation potential by quiescent/cell cycle–arrested aneuploid cells through a re-entry into the cell cycle to contribute to disease progression[148]. Chemotherapy-imposed selective pressures might trigger this response, which exploits intrinsic survival capabilities to finally result in a phenotypic switch toward gaining stem-like characteristics.

One more emerging model of the EOCSC-dependent chemoresistance is their specific expression of the drug membrane transporters[248,249,250]. Multidrug resistance (MDR) can be defined as the ability of cancer cells to acquire resistance to a broad spectrum of structurally and functionally different anticancer drugs, and is thought to be a major obstacle to the success of cancer chemotherapy. It is commonly accepted that MDR is mediated mainly by the overexpression of ATP-binding cassette (ABC) transporters that remove substrate drugs out of the cells against a concentration gradient with the use of energy from ATP hydrolysis[251,252,253]. Forty-eight different ABC transporters have been identified in the human genome and are divided into seven subfamilies (A–G) based on sequence similarities, among which ABCB1/P-glycoprotein, ABCC1/MRP1, and ABCG2/BCRP are the most important members[254,255,256]. Calcagno et al. studied the development of MDR in breast, ovarian, and colon cancer cell lines (MCF-7, IGROV-1, and S-1) mediated by ABC transporters. Surprisingly, a single-step selection with low doses of anticancer agents, similar to the concentrations reported *in vivo*, induced MDR that was mediated exclusively by ABCG2/BCRP1 in the established experimental model. ABCG2 has shown overexpression at a mRNA at protein levels, which was facilitated by epigenetic changes (histone hyperacetylation due to weaker histone deacetylase 1–promoter association)[257]. ABCG2 is a membrane transporter of dye Hoechst 33342 that is commonly used to define and isolate SP cells in several normal mammalian tissues, such as bone marrow, breast, skin, and ovaries, as well as cancers that arise from these tissues[56,133,134,135,136,138]. SP cells from two distinct genetically engineered mouse ovarian cancer cell lines expressed putative stem markers and showed resistance to doxorubicin and paclitaxel due to ABCG2 expression. This SP cell population comprised membrane transporter–expressing putative stem-like cells and was highly tumorigenic in *in vivo* assays compared to non-SP cells[56].

Recently, Ma et al. identified a cancer stem-like and chemoresistant cell subpopulation present in the human EOCSC line SKOV3. Subjecting SKOV3 cell lines to constant concentrations of cisplatin and paclitaxel under the stem cell culture conditions distinguished spherical cells, which displayed stem-like properties and characteristics of drug resistance at the same time. These cells showed a remarkable self-renewal capacity, high tumor-forming ability, and the overexpression of Nanog, Oct4, Sox-2, Nestin, CD133, CD117, and other stem-related genes. This SKOV3 cell subpopulation showed chemoresistance not only to drugs used in the selection protocol, but also to other chemotherapeutic agents, such as adriamycin and methotrexate. Studying alterations of genetic profiles in SKOV3 sphere cells, compared to other SKOV3 cells, revealed 3487 genes with more than a twofold difference in expression, of which approximately 170 genes showed more than a 10-fold difference. Through functional clustering of the differentially expressed genes, a large proportion of the significantly different genes were found to be related to angiogenesis, extracellular matrix, integrin-mediated signaling pathway, cell adhesion, and cell proliferation[250]. The isolation of this stem-like and chemoresistant subpopulation from the SKOV3 cell line might represent a suitable *in vitro* model for identifying and further studying tumorigenic subpopulations of ovarian cancer cell lines under strict experimental conditions *in vitro*.

## CONCLUDING REMARKS

In addition to all described experimental results, there are several characteristics of EOC supporting the view that it should be considered as a stem-based disease. EOCs found on the surface of the ovary display a range of histologic subtypes, which perhaps surprisingly recapitulate the histology of other normal gynecological tissues. The high rate of recurrent disease, after initial treatment success, suggests that there is a small population of tumor cells that have the ability to repopulate the entire tumor burden and that these cells exhibit cytoprotective mechanisms, allowing them to survive and eventually develop chemoresistance.

The appreciation of this new concept will allow for a more rational approach to treatment that can potentially have a significant impact on reducing the mortality of this devastating disease. Novel drug targets may include self-renewing and signaling pathways, intrinsic survival and niche factors, drug transporters, and other surface functional molecules, and novel therapeutic approaches may involve the differentiation of EOCSCs into more drug-sensitive descendants, as well as immunotherapeutic regimes specifically targeting the EOCSC subpopulation.

However, several important issues remain elusive, including the question of the cells of origin for each EOC subtype and the lack of biomarkers for screening and early detection of disease. This task is very difficult, bearing in mind the fact that we are still in the dark when it comes to understanding the etiology of these tumors. More profound studies and better experimental model systems are urgently needed to resolve the significant gaps in the EOC research field. Many current publications purporting to be evidence of certain cellular behaviors are, in fact, derived from cell lines, not primary EOCs, and therefore may not reflect unmanipulated EOC characteristics. Given the heterogeneity of this disease, increases in long-term survival might be achieved by translating recent insights at the molecular and cellular levels to identify individual strategies for treatment and to optimize early detection. The identification of tumor type-specific EOCSC molecular targets shows the future promise of personalized therapy.

## ACKNOWLEDGMENTS

This work was supported by a Grant, No 175092, from the Ministry of Science and Technical Development of Serbia.
